# A Canine Distemper Virus Retrospective Study Conducted from 2011 to 2019 in Central Italy (Latium and Tuscany Regions)

**DOI:** 10.3390/v13020272

**Published:** 2021-02-10

**Authors:** Ida Ricci, Antonella Cersini, Giuseppe Manna, Gaetana Anita Marcario, Raffaella Conti, Giuseppina Brocherel, Goffredo Grifoni, Claudia Eleni, Maria Teresa Scicluna

**Affiliations:** Istituto Zooprofilattico Sperimentale del Lazio e della Toscana “M. Aleandri”, Via Appia Nuova, 1411, 00178 Roma, Italy; giuseppe.manna@izslt.it (G.M.); gaetanaanita.marcario@izslt.it (G.A.M.); raffaella.conti@izslt.it (R.C.); giuseppina.brocherel@izslt.it (G.B.); goffredo.grifoni@izslt.it (G.G.); claudia.eleni@izslt.it (C.E.); teresa.scicluna@izslt.it (M.T.S.)

**Keywords:** canine distemper virus, passive surveillance, Real time PCR, lineage

## Abstract

Canine distemper virus (CDV) is a highly lethal contagious viral pathogen mainly found in domestic and wild canids and mustelids. Although, in Italy, circulating strains of Europe 1, Europe wildlife and Arctic type are reported, data relating to Latium and Tuscany regions are limited. In view of this, through passive surveillance, we investigated the presence of CDV and which strains were circulating in these Regions. From March 2017 to October 2019, a group of 122 subjects were tested for CDV using a PCR protocol described in the literature, with 12 detected positive; analyses were carried out on a set of target samples (brain and lung, conjunctival, nasal and rectal swabs, urine or swab from bladder and intracardiac clot) that was defined for the detection of CDV in both live and dead animals. The rectal swab, easily collected also from live animals, represented the most suitable sample for CDV diagnosis, with 9 positive of the 11 (81.82%) tested. In addition, brain and lung of 15 subjects out of 181 susceptible animals collected between 2011 and 2018, during post mortem investigations in routine diagnostic activity, were CDV positive. Molecular analyses of all positive samples, using a 287 bp fragment located within the conserved N terminus of the morbillivirus nucleoprotein gene, detected the circulation of strain CDV599/2016 (KX545421.1) belonging to the “Europe wildlife” lineage, and of strain CDV12254/2015 (KX024709.1), belonging to the Arctic-lineage, thus confirming the co-circulation of the two lineages, as already noted in previous studies.

## 1. Introduction

Canine distemper virus (CDV) represents one of the main causes of a viral systemic pathology with lethal outcome in domestic and wild carnivores (mainly canids and mustelids), but also in numerous other wild species [1]. The main factors that favor the spread of the virus are genetic variability, the broad host spectrum, and uncontrolled animal movements of stray and domestic dogs. Vaccination still remains the main measure for disease prevention today.

CDV, the etiological agent of Canine Distemper (CD), is a member of the genus Morbillivirus that belongs to the *Paramyxoviridae* family; its genome is a single strand RNA, with negative polarity. Like other paramyxoviruses, the virus contains six structural proteins that are the nucleocapsid (N), matrix (M), fusion (F), hemagglutinin (H), polymerase (L) and phosphoprotein (P) [1,2].

The circulating CDV strains are divided into different groups in relation to their geographical distribution and genetic characteristics, and, in particular, relative to the gene encoding for the H protein. Indeed, the study of the phylogenetic and molecular evolutionary analysis of the CDV revealed that the appearance of the disease in new species is correlated to mutations affecting the H protein-binding site for virus entry receptors [3,4,5]. There are numerous reports of CDV in Italy, describing also the different viral circulating strains belonging to distinct genetic lineages [6,7,8,9]. Some studies additionally report pathogenetic differences between the different strains responsible for CDV infections, such as for tissue tropism inducing an assortment of histopathological lesions [9,10].

In Italy, the availability and administration of effective vaccines allow the control of CDV infections. However, outbreaks still occasionally occur, which are generally related to the illegal trade of infected dogs, especially from Eastern Europe [4,11,12,13]. The last major CDV reported epidemic in Italy occurred in 2013 in Abruzzo, a region characterized by the presence of numerous natural parks with a significant animal biodiversity, and was caused by a strain belonging to the Arctic lineage (prototype CDV2784/2013), detected for the first time in the wild population in Europe [4,5]. In this outbreak, CDV caused clinical disease in unvaccinated domestic dogs, Appennine wolves (*Canis lupus*) and other wild carnivores present in Abruzzo, as well as in the neighboring regions, such as Molise. CDV circulation was also detected in the following years in wild animals of the same area [2,8,13].

Since 2018, an increase in the presence of CDV in foxes (*Vulpes vulpes*) and badgers (*Meles meles*) found dead was detected through passive surveillance in Northern Italy (Region of Friuli Venezia Giulia). 

In Italy, the circulation of strains Europe 1, Europe wildlife and Arctic [4,8,10] has been reported. In this study, we investigated the circulation of CDV, and the characteristics of the circulating strains detected through passive surveillance in the regions of Latium and Tuscany, as these data are not available for these areas. The present study was conducted on wild and domestic animals submitted to Istituto Zooprofilattico Sperimentale del Lazio e della Toscana (IZSLT) for post-mortem examinations between 2011 and 2019, and tests were carried out within a research project funded by the Italian Ministry of Health.

## 2. Materials and Methods

### 2.1. Animals Examined

From March 2017 to October 2019, CDV susceptible wild species found dead or hunted, exotic dead animals of a Tuscany zoo and domestic dogs that died with a clinical history of neurological signs and other symptoms and/or lesions attributable to CD, were submitted to IZSLT for post-mortem examination and sampled for CDV diagnosis within a research project funded by the Ministry of Health; in this period, we received a complete set of biological samples [14] consisting of brain and lung, conjunctival, nasal and rectal swabs but also intracardiac clot and urine or bladder swabs collected from a group of 122 subjects. To obtain a broader picture of the CDV circulating strains, we extended our investigation to 181 similar cases of dogs and wild species submitted to our Institute for post-mortem examination between 2011 and 2018, for which only some of the target specimens (lung and brain) were available stored at −20 °C, that were analysed by PCR for CDV.

### 2.2. Sample Collection and Stastistical Analysis of the Results

Within the research project funded by the Ministry of Health (March 2017–October 2019), a set of samples were collected when possible at necropsy from each subject: brain, lung, conjunctival, nasal and rectal swabs, urine (or swab from bladder) and intracardiac clot.

Portions of brain and lung were immediately fixed in 10% neutral buffered formalin for histological and immunohistochemical analysis. The rest of the samples were stored at −20 °C for CDV diagnosis and subsequent biomolecular analysis. The same organs and swabs were also examined for the presence of CDV vaccine strains [15].

For the retrospective study, samples of brain and lung collected from 2011 to 2018, were submitted to biomolecular analysis; in case of PCR positive cases, fresh sections were cut from brain and lung samples, previously included in paraffin and stained for histological examination and for immunohistochemistry.

Fisher’s test (*p*-value < 0.05) was used [16] to analyse the results obtained for the different biological samples of positive animals to identify the most suitable biological sample for CDV diagnosis.

### 2.3. RNA Extraction

Total RNA was extracted by homogenizing 20–30 mg tissue by using high speed shaking in Eppendorf tubes with stainless steel beads (5 mm diameter in the TissueLyser II (QIAGEN). Homogenates were centrifuged at 20,000× *g* for 5 min at room temperature and 200 µL of the supernatants were submitted to RNA extraction with QIAamp cador Pathogen Mini Kit (QIAGEN) according to the manufacturer’s instructions. The same protocol was employed to extract total RNA from 200 µL of urine and from swabs (200 µL of vortexed 1 mL of transport medium in which the swabs are immersed); medium used for swabs was Dulbecco’s modified Eagle’s medium (D-MEM) added with Fetal Bovine Serum (2%), penicillin (500 IU/mL) and stremptomycin (500 µg/mL). RNA was eluted from each sample in a final volume of 60 µL of elution buffer using QIAgen columns.

### 2.4. Reverse Transcription and cDNA Synthesis

Reverse transcription was carried out using a volume of 30 µL of RNA with the addition of the following reagents: 6 µL of 10× random hexamer primers, 6 µL 10× RT-Buffer, 2.4 µL dNTP mix 100 mM, 3 µL of 5U Multi Scribe Reverse Transcriptase and 12.6 µL of RNase-free water by High Capacity cDNA Reverse Transcription kit (Applied Biosystems™, ThermoFisher Scientific); cDNA synthesis was carried out using Gene Amp^®^ PCR System 9700 (Applied Biosystems) using the following thermal amplification profile: 25 °C for 10 min, 37 °C for 45 min and 85 °C for 5 min.

### 2.5. Real Time PCR for Field CDV Strain Diagnosis

For field CDV strain diagnosis, the specific primer pair (CDVF4 5′-GTCGGTAATCGAGGATTCGAGAG-3′ and CDVR 5′-GCCGAAAGAATATCCCCAGTTAG-3′) and the TaqMan Probe (3CDV 5′-6-FAM-ATCTTCGCCAGAATCCTCAGTGCT-MGB-3′), designed on a highly conserved region of the P gene, were used [17] in the Real Time PCR using the AgPATH-ID™ One-step RT-PCR Kit (Applied Biosystems™, ThermoFisher Scientific) with the following composition: 12.5 µL of 2X RT-PCR Buffer, 1 µL of 25X RT-PCR Enzyme mix, 600 nM of forward primer, 600 nM of reverse primer, 300 nM of probe, 5 µL of RNA and 4.75 µL RNase-free water for a 25 µL total final volume. The Real Time PCR was carried out using System QuantStudio 7 Flex (Applied Biosystems) and the conditions were 45 °C for 10 min, 95 °C for 10 min, and 50 cycles of 95 °C for 15 s, 60° C for 1 min and 10 s. The thermal cycles were optimized following the manufacturer’s instructions.

All data were analysed using the System QuantStudio 7 Flex Sequence Detection System SDS software package (Applied Biosystems, Foster City, CA, USA).

### 2.6. Real Time PCR for Vaccine CDV Strains Diagnosis

For CDV vaccine strain diagnosis, the primer pair used was (CDV Vaccine Fw 5′-ATAATGATGTTATCATCAGYGATGAT-3′ and CDV Vaccine Rv 5′-CTTGGTCCGATAATGATCAACC-3′) together with the TaqMan Probe (CDV probe AM1 5′-FAM-CTTAGTAGCAYTGCCCAAGATCCCTTGATC-BHQ1-3′), designed in a 249 bp portion of the CDV M gene and M-F intergenic region of the Onderstepoort vaccine strain. This Real Time PCR protocol was developed to specifically identify the following vaccine strains: Onderstepoort, Duramune, Snyder Hill, Nobivac [15]. For the Real Time PCR vaccine CDV strains, the TaqMan^®^ Universal Master Mix kit (Applied Biosystems™, ThermoFisher Scientific) was used with the following composition: 12.5 µL of TaqMan^®^ 2X Universal PCR Master Mix, 600 nM of forward primer, 600 nM of reverse primer, 300 nM of probe, 5 µL of cDNA and 5.75 µL RNase-free water for a 25 µL total final volume. The Real Time PCR for vaccine CDV strains was carried out using Quant Studio 7 Flex (Applied Biosystems) and the conditions were: 50 °C for 2 min, 95 °C for 10 min, and 50 cycles of 95 °C for 15 s, 58 °C for 30 s and 60 °C for 1 min. All data were analysed using the Quant Studio 7 Flex Detection System SDS software package (Applied Biosystems, Foster City, CA, USA). All the examined samples were also tested in parallel with the Wilkes protocol to exclude any positivity from being linked to vaccinations.

### 2.7. PCR for the CDV Strains Characterization

For the samples resulting positive in Real Time PCR, we proceeded with the genetic characterization of the CDV strain. The PCR protocol amplified a 287 bp fragment located on the conserved N terminus of the morbillivirus NP gene and used primers (MvF 5′-ACAGGATTGCKGAGGACCTAT-3′ and MvR 5′-VARGATAACCATGTACGGTGC-3′) degenerated in position 779 (forward primer) and 1055 and 1053 (reverse primer), respectively [18]. The Master Mix was carried out on a final volume of 50 µL and consisted of: 5 µL Platinum Taq Buffer 10×, 2 µL dNTPmix 10 mM (at 0.4 mM final concentration), 2.5 µL MgCl2 50 mM (at 2.5 mM final concentration), 0.33 µL for both the MvF 30 µM and MvR30 µM (each to the final concentration of 0.2 µM), 0.5 µL Platinum™ Taq DNA Polymerase 5 U/µL (Invitrogen™, ThermoFisher Scientific) (at 0.05 U/µL final concentration), 5 µL cDNA template and 34.34 µL of RNase-free water. The reaction was performed in a Gene Amp^®^ PCR System 9700 (Applied Biosystems) with the following PCR conditions: 95 °C for 10 min, 40 cycles of 95 °C for 1 min, 59.5 °C for 1 min, 72 °C for 1 min; 72 °C for 10 min. PCR products were visualized in 1.5% agarose gel electrophoresis by staining with GelRed dye (Biotium, Hayward, CA, USA).

### 2.8. Sequencing for CDV Strain Characterization

The amplicons for CDV strain characterization were purified with the QIAquick PCR Purification kit (Qiagen, Hilden, Germany) and sequenced using the PCR primers MvF/MvR as described in point 2.7 with the BigDye Terminator Cycle Sequencing Ready Reaction kit, version 3.1 (PerkinElmer, Applied Biosystems, Foster City, CA, USA) in an automated sequencer (ABI Prism 310 DNA sequencer, Applied Biosystems, Foster City, CA, USA). Alignments were obtained with the multiple program DNASTAR (DNASTAR Inc., Madison, WI, USA) using Clustal W. The nucleotide sequences obtained were analysed using Basic Local Alignment Search Tool (BLAST) by comparing them to sequences from reference strains of different CDV virus accessed on NCBI GenBank (http://www.ncbi.nlm.nih.gov/ (accessed on April 2020). The samples were considered as belonging to a particular strain when sequence identity and query cover were between 98 and 100%.

### 2.9. Histological and Immunohistochemical Exams

Portions of brain and lung of PCR positive animals, previously fixed in formalin, were embedded in paraffin, cut at thickness of 5 µm and routinely stained with hematoxylin and eosin.

Immunohistochemistry was carried out on newly prepared unstained sections of both organs, using the following procedure: blocking of endogenous peroxidase with 3% H_2_O_2_ at room temperature for 30 min, antigen retrieving with trypsin at 37 °C for 30 min, overnight incubation at 4 °C with a monoclonal antibody anti-Canine Distemper Virus (clone 8-1) (LSBio). A positive reaction was detected using 3, 3′- diaminobenzidine (EnVision Plus kit, Dako) as chromogen with a 3-min development at room temperature and counterstained with hematoxylin.

## 3. Results

### 3.1. Animals Examined

A total of 303 subjects received from 2011 to 2019 were tested for CDV using a PCR protocol; their distribution by species is shown in Table 1. Over the entire period, dog (*Canis lupus familiaris*), fox (*Vulpes vulpes*) and wolf (*Canis lupus*) were the most frequent species representing, respectively, 35.97% (109/303), 36.63% (111/303) and 15.18% (46/303) of the total subjects tested.

Table 1 reports the total distribution by species of all examined and positive subjects detected from 2011 to 2019, as well as relative percentages.

### 3.2. Diagnostic Test Results

The samples used to obtain the results presented in Table 1 and Table 2 are described below. A total of 27 animals, of the 303 examined, were diagnosed as positive: their distribution by species is shown in Table 1.

Twelve subjects were detected positive out of the 122 animals of which we received a complete set of biological samples collected from March 2017 to October 2019, equal to 9.84% (12/122). As represented in Table 2, for this group, the positive subjects were eight foxes (66.66%), two dogs (16.67%) and two wolves (16.67%); five subjects were from the province of Arezzo (four foxes and one wolf), four from Rome (three foxes and one dog) and three from Rieti (one dog, one fox and one wolf). Relative to the 181 subjects received from 2011 to 2018 for post-mortem investigation, of which brain and lung were available, 15 were detected as positive for CDV, equal to 8.29% (15/181) (Table 2). Among these there were 10 dogs (66.66%), four foxes (26.67%) and one wolf (6.67%); two subjects came from province of Frosinone (two dogs), one from Pistoia (one dog), one from Rieti (one wolf), eight from Rome (four dogs and four foxes), two from Siena (two dogs) and one from Viterbo (one dog). All positive samples were also tested in parallel with the Wilkes protocol to exclude any positivity from being linked to vaccination [15], with all testing negative. The overall distribution of positive subjects by province and species relative to 2011–2019 is shown in Table 2.

Relative to the group of 122 subjects, of which it was possible to examine a complete set of biological samples, the following number of samples were examined: 111 brains, 117 lungs, 96 conjunctival swabs, 95 nasal swabs, 91 rectal swabs, 46 intracardiac clot, 48 bladder swabs and 32 urine samples.

For these subjects, analysing the results obtained with the molecular tests on the different samples of the positive animals, it was observed that a positive lung and/or brain outcome (standard routine biological sample used so far for post-mortem molecular diagnosis) corresponds to at least one positive result among the conjunctival, nasal or rectal swabs, samples easily obtainable in live animals and representing a very good opportunity for in vivo diagnosis. The opposite was not always the case for subjects with positive results in only one or more swabs, as these did not constantly show CDV-positive results in the brain and/or lung samples, thus confirming the importance of these biological samples for diagnosis in live animals, as already observed by other authors [19].

Table 3 reports the detailed results of the molecular tests including the cycles (Ct) obtained in Real Time PCR; samples with Ct values ≤38 were considered positive for CDV based on sensitivity studies performed with field samples and synthetic targets. Of the 12 total positive subjects, one was excluded from the comparison since not all of the biological samples used for the comparative analyses were used (brain, lung, conjunctival, nasal and rectal swab). From the analysis carried out with the Fisher’s test with a *p*-value < 0.05, no statistical differences emerged between the different biological samples for the positive subjects (Table 4), even if in comparing the results obtained for the samples available in all 11 subjects, the rectal swab seems to be the most suitable biological sample for in vivo diagnosis, with a number of positive outcomes equal to 9/11 and a percentage approximately equal to 81.82% (Table 3), detecting one positive subject more than the conjunctival and nasal swabs (8/11), equivalent to 72.73%, and 3 more than lung (6/11), equivalent to 54.55%.

#### 3.2.1. Histological and Immunohistochemical Results

Microscopic lung lesions were found only in one positive dog and were represented by mild multifocal interstitial pneumonia, with few lymphocytic infiltrates in the septa and small aggregates of macrophages in the lumen of the alveoli. No specific lesions were detected in the other cases, which could also be due to the frequent bad state of conservation of the examined carcasses or the presence of severe lesions caused by cardiopulmonary parasites.

In the dog, brain lesions were mainly characterized by white matter demyelination and multifocal necrosis of the neurons of the cerebral cortex and of the ependymal cells of the lateral ventricles. In one of the positive foxes, mild ependymitis and multifocal lymphomonocytic meningitis were seen. No significant lesions were observed in the other cases.

**Figure 1:**
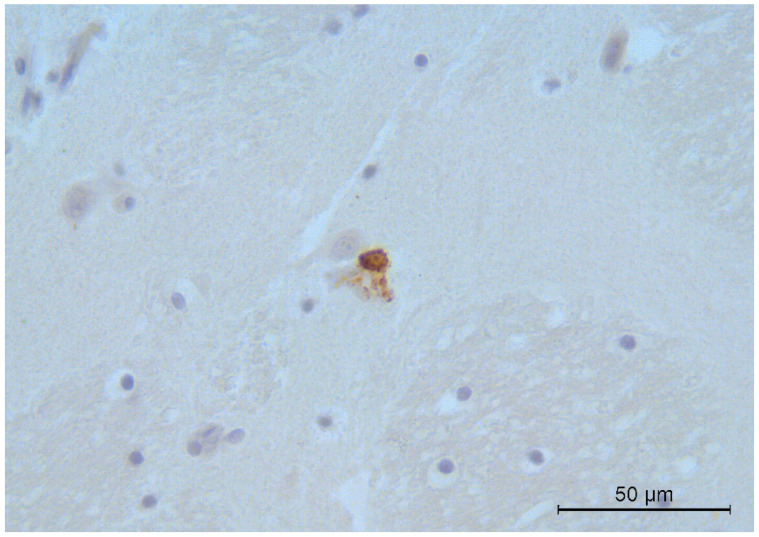
Fox. Brain. Intense CDV immunoreactivity in a cerebral cortex neuron. Immunohistochemistry, 400× magnification.

Immunohistochemical analysis showed specific CDV immune reactivity in alveolar epithelial cells and in the alveolar macrophages of one fox with the other animals testing negative. All brain samples examined were weakly positive, except for the dog. Positivity was multifocally observed in the neurons (Figure 1), especially of the cerebral cortex, thalamus, and brain stem. Few positively stained mononuclear cells were also observed within the meninges and ependyma.

#### 3.2.2. Genetic Analysis of the NP Gene

In this study, the molecular analyses detected the following strains:

(1) One with 100% identity and query cover with the strain CDV 599/2016, Accession Number KX545421.1, that belongs to the “Europe wildlife” lineage [5,8];
(2) A second with 98% identity and query cover with the strain CDV12254/2015, Accession Number KX024709.1 [8], that belongs to the “Arctic” lineage [5,8,20];
(3) A third with 100% identity and query cover with the Canine Morbillivirus strain isolated BJ16C9, Accession Number MF926604.1, that is part of the cluster of America-1 CDV strains closely related to the Onderstepoort vaccine strain [15,20]; the former was isolated only in the lung and rectal swab of a dog in the province of Rome.

A summary of the identified strains is shown in Table 5, indicating the species and the year in which the analysed subject was received.

**Figure 2:**
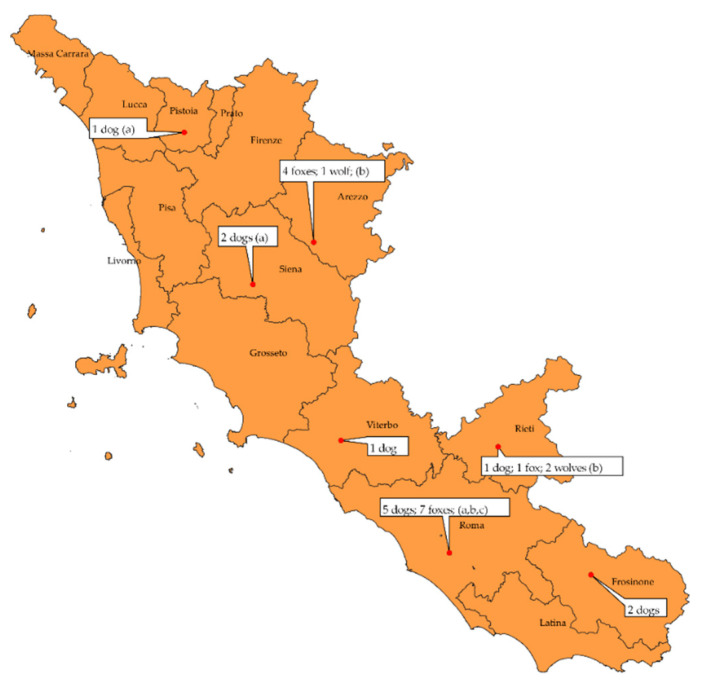
Distribution of positive animals by species and province of origin and identified strains. Strains detected: a: CDV 12254/2015; b: CDV 599/2016; c: BJ 16C9.

Furthermore, Figure 2 shows a map with the species and origin of the positive subjects, including the identified strains.

## 4. Discussion

The analysis of the detected sequences, starting from the samples received at the IZSLT for diagnostic purposes from 2011 to 2019, highlighted the circulation of the strain CDV 12254/2015 (KX024709.1) belonging to the “Arctic” lineage [20] in dogs coming from provinces of Latium and Tuscany. Starting from 2017, genetic analyses conducted on samples from wild animals (fox and wolf) received at our Institute for diagnostic or research purposes detected the circulation, in Latium and Tuscany regions, of the strain CDV599/2016 (KX545421.1) belonging to the “Europe wildlife” lineage, and, in a dog coming from Rome, of strain CDV/BJ16C9 (MF926604.1). These outcomes confirm the co-circulation of the “Europe wildlife” and “Arctic” lineages in Central Italy, as already noted by Di Sabatino [4], while, in Italy, the circulation of at least three separate CDV lineages was confirmed by other authors, including Europa-1 [8], which has not yet been detected in our area of study.

The circulation of the Arctic strain in wild animals already reported in Central Italy highlights the possible role of reservoir that these species can have, with the possibility of passing the strain to unvaccinated domestic animals and the occurrence of subsequent epidemic events. The data obtained in the study carried out indicate two different and separate cycles for CDV distribution in wild and in domestic animals in the area under study, and the characterization of different strains in the two different populations leads to the hypothesis that, for these Regions, there exist distinct epidemiological cycles that are kept separated by ecological barriers.

To date, the CDV/BJ16C9 strain (MF926604.1) that belongs to the cluster of America-1 CDV strains closely related to the Onderstepoort vaccine strain [21,22,23] is the first report of its circulation in our territory. As none of the samples tested positive to the Wilkes protocol [15], the hypotheses explaining the presence of this vaccine strain could be: (a) an antigenic escape; (b) a genetic recombination with wild-type strains; (c) an environmental adaptation; (d) an evolution of the CDV [24]. The small number of positives identified requires further investigation, which we are carrying out to identify more positive subjects and characterize their strains, to strengthen the data obtained and to confirm the simultaneous circulation in the area under study of different strains in wild and domestic populations.

The animals detected positive were dogs, foxes and wolves, which are, however, the most represented species. Not having had a relevant number of positive subjects, it was impossible for us to define the histolesive characterization of the isolated strains.

The importance of taking the complete set of conjunctival, nasal and rectal swabs to maximize the sensitivity of the diagnostic system in the event of a suspicion of distemper in both living and dead subjects—associated also, in the latter case, with the brain and lung—is highlighted. In particular, the rectal swab is apparently the most appropriate sample, but due to the limited number of positive subjects, further samples are required to confirm this.

It will be interesting to continue to monitor domestic dogs and other wild species with symptoms attributable to CDV and verify the different circulating field strains in central Italy, which could provide important information on the evolution of the virus and employing different vaccine strains. The use of the two PCR methods selected for the respective detection of CDV field and vaccine strains allows the advantage of being able to exclude positivity related to vaccination.

For the genetic characterization, the NP gene, as also employed by other authors [25,26,27], was chosen because it contains a conserved region that allows the detection of the known CDV strains circulating in mammals (wild and otherwise). The choice of the NP gene for identification and sequencing of the strains was made by selecting a region of the nucleoprotein (NP) of the CDV, which also shows a great homology among morbillivirus strains, as reported by Verna [18] in reference to previous papers in which the NP region appears as an appropriate candidate for the analysis of circulating strains of CDV [28,29]. The genetic study of the circulating strains by the laboratories that carry out the diagnosis provides information on the evolution of the virus that could be useful for updating vaccine strains. Collaboration between laboratories and vaccine manufacturers would be desirable to produce effective immunization products.

## Figures and Tables

**Table 1:** Distribution of positive subjects by species, collected between 2011 and 2019.

SPECIES	N° Subjects	%/Total	N° PCR Positive	% Positivity
Badger (*Meles meles*)	18	5.94	0	0
Beech marten (*Martes foina*)	1	0.33	0	0
Dog (*Canis lupus familiaris*)	109	35.97	12	44.44
Ferret (*Mustela putorius furo*)	4	1.32	0	0
Fox (*Vulpes vulpes*)	111	36.63	12	44.44
Marmot (*Marmota marmota*)	1	0.33	0	0
Marten (*Martes martes*)	3	0.99	0	0
Mongoose (*Herpestidae*) *	1	0.33	0	0
Crested porcupine (*Hystrix cristata*)	2	0.67	0	0
Raccoon (*Procyon lotor*)	6	1.98	0	0
Squirrel (*Sciurus vulgaris*)	1	0.33	0	0
Wolf (*Canis lupus*)	46	15.18	3	11.12
**Total n°**	303	100	27	100

* Animal held inside a zoo.

**Table 2:** Distribution of positive subjects by province and species (2011–2019).

Province	2017–2019 (Research Project)			2011–2018 (Retrospective Study)			Total
	Dog	Fox	Wolf	Dog	Fox	Wolf	
**Arezzo**		4	1				**5**
**Frosinone**				2			**2**
**Pistoia**				1			**1**
**Rieti**	1	1	1			1	**4**
**Rome**	1	3		4	4		**12**
**Siena**				2			**2**
**Viterbo**				**1**			**1**
**Total**	**2**	**8**	**2**	**10**	**4**	**1**	**27**

**Table 3:** Results obtained with Real Time PCR for the different samples of positive subjects relative to 2017–2019 (Ct).

	Intracardiac Clot	Brain	Lung	Conjunctival Swab	Nasal Swab	Rectal Swab	Bladder Swab/Urine
	neg	neg	neg	35	35	36	35
	n.r. *	37	neg	neg	36	36	n.r. *
	neg	neg	38	neg	38	neg	n.r. *
	n.r. *	28	25	20	20	19	16
	n.r. *	neg	37	36	38	35	36
	n.r. *	neg	neg	34	neg	35	n.r. *
	26	24	19	17	16	17	16
	neg	35	neg	38	neg	neg	neg
	neg	neg	neg	34	38	38	neg
	n.r. *	neg	37	neg	neg	27	neg
	n.r. *	27	34	31	27	34	34
**N° sample**	5	11	11	11	11	11	8
**N° positive**	1	5	6	8	8	9	5
**% positive**	20.00	45.45	54.55	72.73	72.73	81.82	62.50

neg: negative sample; * n.r.: sample not received.

**Table 4:** Values obtained for the comparison of the positivity of the biological samples with the Fisher’s test (*p*-value < 0.05).

SAMPLE	Bladder Swab/Urine	Brain	Conjunctival Swab	Intracardiac Clot	Lung	Nasal Swab	Rectal Swab
**Rectal swab**	0.57	0.18	1	0.52	0.36	1	
**Nasal swab**	1	0.39	1	0.21	0.66		
**Lung**	1	1	0.66	1			
**Intracardiac clot**	1	1	0.21				
**Conjunctival swab**	0.57	0.39					
**Brain**	1						
**Bladder swab/Urine**							

**Table 5:** Strains identified for the positive subjects collected between 2011 and 2019.

Year	Province	Species	N° Subject	Sequence *	Lineage
2013	Rome	Dog	1	Canine distemper virus isolate CDV12254/2015. Query cover 98%. Identity 98%. GenBank: KX024709.1	Arctic
2014	Rome	Dog	2		
	Pistoia		1		
2015	Siena	Dog	1		
2016			1		
2017	Rieti	Fox	1	Canine distemper virus isolate CDV599/2016, partial genome. Query cover 100%. Identity 100%. GenBank: KX545421.1	Europe wildlife
2018	Arezzo	Wolf	1		
	Rome	Fox	1		
	Rome		1		
2019	Rome	Dog	1	Canine morbillivirus isolate BJ16C9, complete genome. Query cover 100%. Identity 100%. GenBank: MF926604.1	America-1

* Sequence investigated: NP gene (287 bp).

## Data Availability

Not applicable.
